# Anterior Cruciate Ligament Reconstruction in Ehlers-Danlos Syndrome

**DOI:** 10.1155/2015/160381

**Published:** 2015-06-28

**Authors:** John Williams, Jonathan Hutt, Mark Rickman

**Affiliations:** Trauma and Orthopaedic Department, St George's University Hospital, London SW17 0QT, UK

## Abstract

This report details the reconstruction of the anterior cruciate ligament in an 18-year-old man with Ehlers-Danlos syndrome (EDS). The reduced mechanical properties of the tissue in EDS can pose a challenge to the orthopaedic surgeon. In this case, we describe the use of a hamstring autograft combined with a Ligament Advanced Reinforcement System (LARS). There was a good radiographical, clinical, and functional outcome after two years. This technique gave a successful outcome in the reconstruction of the ACL in a patient with EDS and therefore may help surgeons faced with the same clinical scenario.

## 1. Introduction

Ehlers-Danlos syndrome (EDS) is a heterogeneous group of disorders characterised by joint hypermobility and fragile, hyperextensile tissues. It affects around 1 in 5000 live births, and orthopaedic manifestations especially involving the knee and shoulder joints are common [1]. In EDS, ligamentous and capsular laxity in the knee can cause recurrent dislocation and subluxation, leading to bony and soft tissue damage necessitating surgical repair or reconstruction of ligaments [1, 2]. Repair alone presents difficulties due to the reduced mechanical properties of the tissue, which also restricts the use of autogenous grafts for reconstruction. The use of allografts or synthetic materials is another option.

Rupture of the anterior cruciate ligament (ACL) is a common sports injury [3]. Surgical reconstruction may be indicated in symptomatic or high-demand patients and a variety of grafts are available. These may be autografts, allografts, or synthetic materials. One popular example of the latter is the Ligament Advanced Reinforcement System (LARS), a nonabsorbable synthetic ligament made from terephthalic polyethylene polyester fibres [4, 5]. It is designed for use in conjunction with well vascularised remnants to allow tissue in-growth or as reinforcement alongside autologous grafts. Published reports of restoration of near normal laxity and low rupture rate are encouraging [6].

This case report describes the use of a combined autogenous hamstring and LARS ligament graft to reconstruct the ACL in an EDS patient. We believe this is a novel technique in this setting. The patient has provided consent for publication of the case.

## 2. Case Presentation

**Figure 1:**
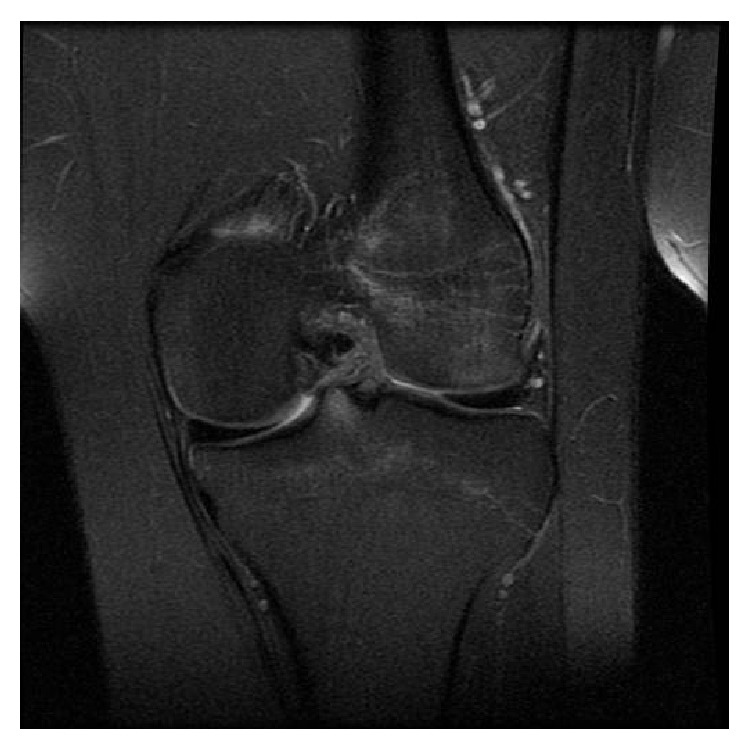
Preoperative coronal MRI scan of the left knee demonstrating a full thickness tear of the anterior cruciate ligament with a typical pattern of bone bruising of the lateral femoral condyle and posterior aspect of the lateral tibial condyle.

**Figure 2:**
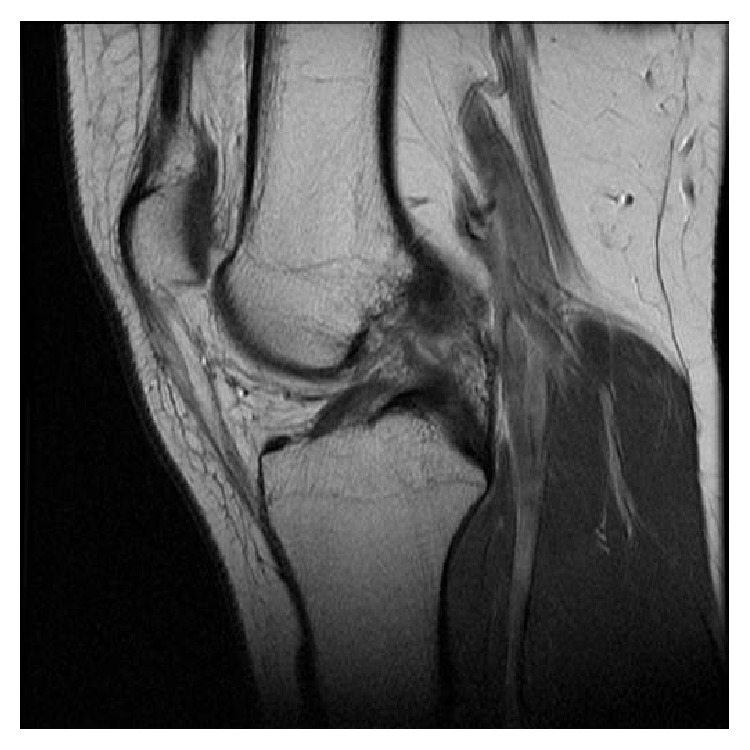
Preoperative sagittal MRI scan of the left knee.

An active eighteen-year-old man presented to his family doctor in December 2011 with a swollen and painful left knee following a twisting injury sustained playing in an amateur soccer game. This patient had no previous injuries to the knee and was otherwise medically fit, with a known diagnosis of EDS. He had a MRI scan in February 2012 which demonstrated a full thickness tear of the ACL, with a typical pattern of bone bruising of the lateral femoral condyle and posterior aspect of the lateral tibial condyle (Figures 1 and 2). The posterior cruciate and collateral ligaments were normal. He was subsequently reviewed in the senior author's clinic in April 2012. At this point, the patient had received no treatment and had symptomatic instability and occasional pain. Clinically, the knee had marked anteroposterior laxity. Following discussion, and in light of his significant symptomatic instability and high level of preoperative activity, the option of surgical reconstruction was chosen.

In July 2012, he underwent an arthroscopic ACL reconstruction along with a notchplasty. The notchplasty was performed because hyperextension of the knee in EDS is more common and thus may cause impingement of graft against notch. Additionally, a tight notch may have been the reason for failure of the original ACL. The graft was a combined ipsilateral hamstring autograft (two strands) and LARS ligament (two strands), measuring 9 mm in diameter with endobutton suspensory fixation proximally and interference screw fixation distally after tensioning. Postoperative laxity and pivot shift correction were excellent. Standard physiotherapy protocols were followed.

**Figure 3:**
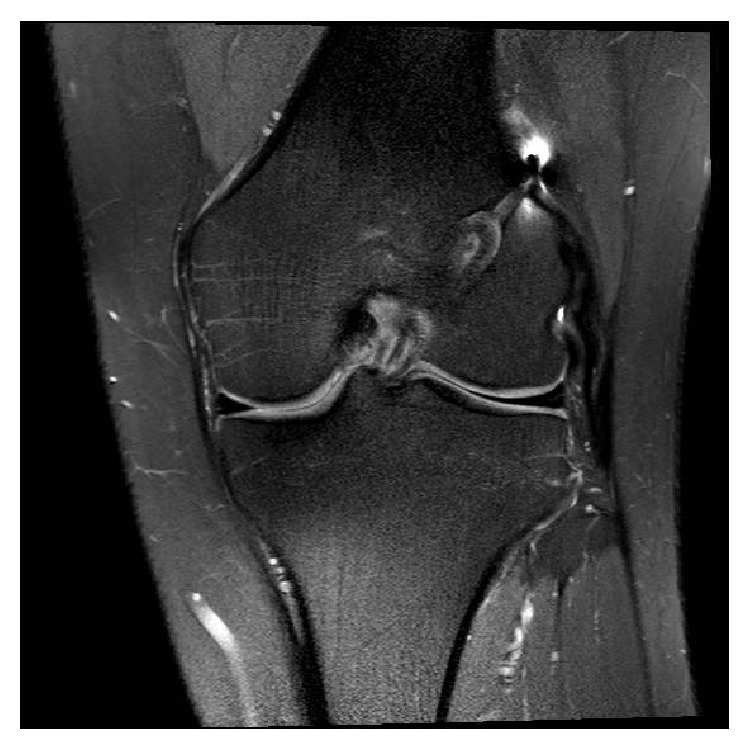
Postoperative coronal MRI scan of the left knee at 16 months demonstrating an intact, well-tensioned reconstruction with no signs of complications.

**Figure 4:**
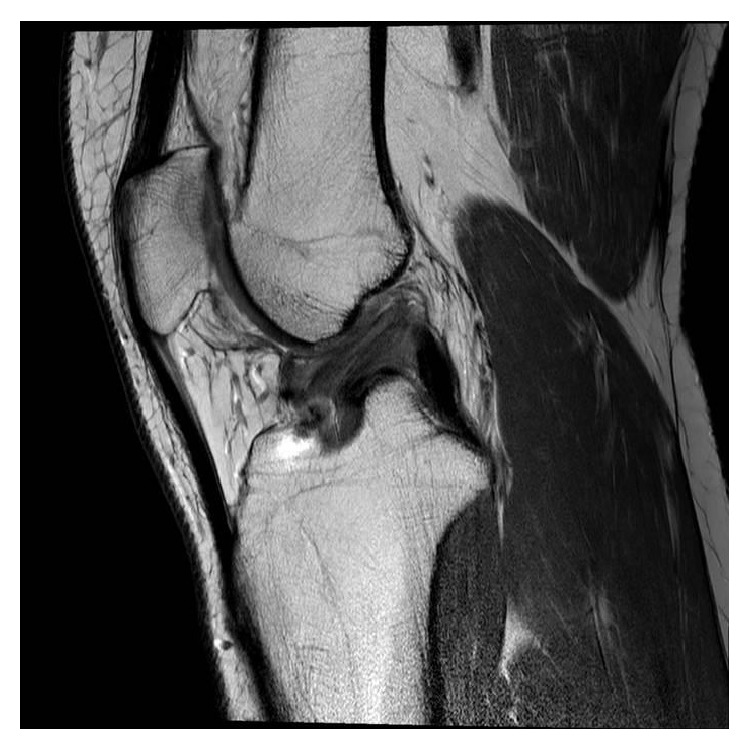
Postoperative sagittal MRI scan of the left knee at 16 months.

At one year after reconstruction his knee on examination was stable and he had resumed sporting activities. MRI scan at 16 months showed the reconstruction to be intact, well-tensioned and with no signs of complications (Figures 3 and 4). Two years postoperatively, the patient scored 90 out of 100 on the Lysholm Knee Scale, and he has returned to playing soccer, tennis, and cricket.

## 3. Discussion

To the best of our knowledge there are no reports of the results of ACL reconstruction in EDS, but there are several reports regarding other ligamentous injuries. A recent paper details the successful use of an allograft Achilles tendon to repair chronic shoulder instability in an EDS patient [7]. Iacono et al. described good results with the reconstruction of a chronic patellar tendon rupture with an allograft alone [8]. Matziolis et al. described successful reconstruction of a patellar tendon rupture in an EDS patient using allograft plus a synthetic ligament for augmentation [9].

However, there are a number of reports of ACL reconstruction in patients with excessive joint laxity. The prevalence of ACL rupture has been reported as higher in patients with excessive joint laxity [10]. Additionally, these patients are more likely to experience poorer outcomes after reconstruction [11, 12]. In this group of patients, the results of ACL reconstruction with a bone-patellar tendon-bone graft have been reported as being superior to those associated with the use of a four-bundle hamstring graft [13]. This result may well be due to the inherent laxity of autograft tissue.

The senior author undertook reconstruction in this case with a combined hamstring/LARS ligament graft because of concerns regarding the ability of potentially weak autograft tissue to provide sufficient stability in a young active man. Hamido et al. (2011) reported that the use of LARS to augment short or undersized allografts is an effective and safe way of improving outcomes, especially when return to sporting activity is required [14], although they did not describe the technique with weakened grafts. Liu et al. retrospectively compared outcomes of LARS ligament and hamstring autograft in 60 patients and found the LARS group displayed nearly normal laxity [15].

## 4. Conclusions

This report details a novel technique for ACL reconstruction in EDS using an autograft augmented with a synthetic ligament with a good outcome radiographically, clinically, and functionally. We believe that this technique may be of benefit in other patients with EDS and ACL rupture to overcome the known challenges of ligament reconstruction in this cohort.
